# Research on the Influence Mechanism of Agricultural Entrepreneurship: Evidence From Five Provinces in Western China

**DOI:** 10.3389/fpsyg.2022.864226

**Published:** 2022-06-01

**Authors:** Hao Dong, Bo Wang, Panpan Zhang, Ximeng Chen, Jichang Han

**Affiliations:** ^1^Shaanxi Provincial Land Engineering Construction Group Co., Ltd., Xi’an, China; ^2^Institute of Land Engineering and Technology, Shaanxi Provincial Land Engineering Construction Group Co., Ltd., Xi’an, China; ^3^Institute of Vocational Education, Nanning Normal University, Nanning, China

**Keywords:** entrepreneurial attitude, entrepreneurial environment, entrepreneurial self-efficacy, entrepreneurial behavior, structural equation model

## Abstract

Individual entrepreneurial behaviors will be affected by their attitudes and environmental factors. Therefore, entrepreneurial attitude and entrepreneurial environment interpret the entrepreneurial behavior mechanism of farmers from the perspectives of internal and external factors. This manuscript is based on a survey data of farmers in five western provinces in China. Using structural equation modeling, the mechanism of the effects of farmers’ entrepreneurial attitudes and entrepreneurial environment on entrepreneurial behavior was analyzed empirically. The research results show that individual entrepreneurial attitudes and the external entrepreneurial environment cannot directly affect entrepreneurial behavior, and both will be through entrepreneurial self-efficacy. The sense of entrepreneurship indirectly affects entrepreneurial behavior, and entrepreneurial self-efficacy plays an intermediary role. The “entrepreneurial behavior structure” model proposed in this manuscript points out that cultivating entrepreneurial attitudes and creating an entrepreneurial environment cannot directly promote entrepreneurial behavior of farmers, but must stimulate farmers’ entrepreneurial behavior. The sense of self-efficacy provides a direction for the promotion of policy formulation and practical guidance for farmers’ entrepreneurship.

## Introduction

The essence of farmers’ entrepreneurship is to match different elements such as resources and opportunities to achieve dynamic balance under the combined effect of internal and external factors (Bouichou et al., 2021). How to promote farmers’ entrepreneurship has become the core issue of farmers’ reform at this stage (Saridakis et al., 2021). The reason why farmers’ entrepreneurship is different from ordinary entrepreneurial activities lies in the peculiarities of farmers’ entrepreneurs, the rural environment, and the heterogeneity of the dynamic capabilities formed by the interaction between the two (Piras et al., 2021). In the process of entrepreneurship, farmers’ characteristics, organizational regulations, and entrepreneurial environment can have a driving effect on entrepreneurial behavior (Bao et al., 2020). Previous scholars have showed that the general external environment, family environment, and personal qualities have a greater impact on farmers’ entrepreneurial behavior (Brush et al., 2008; Liao and Welsch, 2008). The key entrepreneurial behavior constitutes entrepreneurial events (Barbosa et al., 2007; Barakat et al., 2014), and the entrepreneur’s self-efficacy, social background, and economic conditions will trigger the entrepreneurial behaviors, where entrepreneurial self-efficacy is an individual’s subjective level of confidence in his or her ability to achieve entrepreneurial goals (Sequeira et al., 2007). Empirical research showed that different dimensions of entrepreneurial self-efficacy played a partial mediated and fully mediated role between the independent personality and entrepreneurial intention of migrant workers (Chen et al., 1998; Wilson et al., 2007; Kasouf et al., 2015; Tantawy et al., 2021). However, most studies on entrepreneurial self-efficacy have focused on college students and corporate entrepreneurship (Huang et al., 2021; Liu and Yu, 2021; Zelin et al., 2021), and, however, few scholars have studied farmers’ entrepreneurship in the context of rural revitalization in China. The article constructs a mechanistic model of farmers’ entrepreneurial behavior from the perspectives of entrepreneurial attitude and entrepreneurial environment, and uses a structural equation modeling approach to test it empirically in order to provide empirical support for the complex mechanisms among entrepreneurial attitude, entrepreneurial environment, entrepreneurial self-efficacy, and farmers’ entrepreneurial behavior.

## Theoretical Analysis and Research Hypotheses

### The Influence of Entrepreneurial Attitude on Entrepreneurial Behavior

Individual entrepreneurial attitudes are influenced by individual characteristics. Positive entrepreneurial attitudes make individuals confident in entrepreneurship, have high expectations for completing entrepreneurial behavior, and have a high sense of entrepreneurial self-efficacy. The well-known theory of planned behavior has been studied to confirm that entrepreneurial attitudes, subjective norms, and perceptual behavioral attitudes have a direct effect on entrepreneurial behavior and an indirect effect on entrepreneurial behavior through entrepreneurial intentions. Other researchers have come to similar conclusions that the stronger the entrepreneurial attitude of an individual, the stronger the entrepreneurial intention (Kim and Hall, 2021), which in turn affects entrepreneurial behavior. Empirical research also confirmed through empirical research that entrepreneurial attitudes have direct and indirect effects on entrepreneurial behavior (Wilson et al., 2009). Entrepreneurial attitude has a direct impact on entrepreneurial intention, which in turn affects entrepreneurial behavior, so it can be inferred that positive entrepreneurial attitudes have a positive impact on entrepreneurial behavior (Lindblom et al., 2020). Accordingly, the following research hypothesis is proposed:

**Hypothesis (H1).** Entrepreneurial attitude is positively related to entrepreneurial behavior.

### The Influence of Entrepreneurial Environment on Entrepreneurial Behavior

The entrepreneurial environment refers to the external environment in which farmers’ entrepreneurship is located. The article measures five dimensions: policy support environment, socio-economic environment, scientific and cultural environment, financial service environment, and infrastructure environment. Domestic and foreign scholars’ studies on the relationship between entrepreneurial environment and farmers’ entrepreneurial behavior have mainly focused on political, socio-cultural, economic, and other environmental subjects. The more favorable the local policy is to entrepreneurship, the more rapid the economic development, and the more supportive the cultural background is to entrepreneurship, then farmers will generate entrepreneurial behavior. Farmer entrepreneurs gradually transition from passively receiving policy resource support to subjective demand and evaluation of policies (López et al., 2019). Pricinã (2012) focused on the impact of multidimensional entrepreneurial environment on entrepreneurial behavior by studying the impact of economic goals and traditional culture on farmers’ entrepreneurial behavior under social and economic changes in Romania. Okhomina (2010) showed that individual personality traits are related to entrepreneurial behavior and entrepreneurial environment has an impact on the relationship between the two and it can be hypothesized that entrepreneurial environment has an impact on entrepreneurial behavior. Other researchers studied infrastructure environment, government policy environment, industrial development environment, and financial environment can have a direct impact on entrepreneurial behavior of entrepreneurs. Chandrasekaran (2021) stated that infrastructure environment, government policy environment, industrial development environment, and financial environment can have a direct impact on entrepreneurial behavior of entrepreneurs. Kangogo et al. (2020) showed that the institutional environment affects entrepreneurial behavior through a combination of internal and external effects. It can be seen that a favorable entrepreneurial environment positively affects entrepreneurial behavior. Based on this, the following hypothesis is proposed.

**Hypothesis (H2).** Entrepreneurial environment is positively related to entrepreneurial behavior.

### The Role of Entrepreneurial Self-Efficacy

Entrepreneurship self-efficacy refers to the individual’s confidence and belief in accomplishing entrepreneurial goals, measured in terms of confidence in completing specific entrepreneurial content and confidence in completing the entrepreneurial process, respectively. Entrepreneurship attitude and entrepreneurial environment have a positive effect on entrepreneurial self-efficacy, and entrepreneurial self-efficacy contributes to the generation of entrepreneurial behavior. Entrepreneurship self-efficacy can be influenced by individual entrepreneurial attitudes, which generally vary individually due to individual traits. Entrepreneurial attitudes are individual factors and individuals with personality differences will have different entrepreneurial attitudes (Newbery et al., 2018), resulting in different entrepreneurial self-efficacy. The above results were obtained in the studies of Forbes (2005) and Wilson et al. (2007). An individual’s skill level and entrepreneurial experience and entrepreneurial learning can have an impact on entrepreneurial attitudes and thus act on entrepreneurial self-efficacy (Krueger and Brazeal, 1994). Due to individual differences resulting in personality traits generally innovative, risk-taking and proactive, entrepreneurs with different personality traits necessarily hold different attitudes towards entrepreneurship and have different entrepreneurial self-efficacy (Kautonen et al., 2015). This shows that entrepreneurial attitudes can positively influence entrepreneurial self-efficacy.

When the entrepreneurial environment facilitates entrepreneurship and has a positive effect on entrepreneurship, farmers will have confidence in their ability to solve problems in the subsequent entrepreneurial environment and will have a higher sense of entrepreneurial self-efficacy, which is conducive to the implementation of entrepreneurial behavior. The entrepreneurial environment also influences other factors through entrepreneurial self-efficacy. Oh et al. (2020) showed that entrepreneurial environment and entrepreneurial self-efficacy promote entrepreneurial intentions, and entrepreneurial intentions indirectly cause entrepreneurial behavior, so entrepreneurial self-efficacy promotes entrepreneurial behavior (Oh et al., 2020). There is a significant positive effect of entrepreneurial environment on entrepreneurial self-efficacy, and entrepreneurial self-efficacy mediates between entrepreneurial environment and entrepreneurial intentions (Morgan et al., 2010). Martin and Novicevic (2010) showed that the external environment indirectly acts on entrepreneurial performance through entrepreneurial self-efficacy. Barbosa et al. (2007) showed that the optimization of the external environment has a facilitating effect on entrepreneurial self-efficacy. This shows that the entrepreneurial environment positively influences entrepreneurial self-efficacy.

Entrepreneurship self-efficacy determines the strength of entrepreneurial intention and consequently triggers entrepreneurial behavior. Combining the characteristics of the new generation of farmers, entrepreneurial self-efficacy can be divided into three categories: opportunity recognition efficacy, relationship efficacy, and risk tolerance efficacy (Barbosa et al., 2007). The higher the sense of opportunity recognition efficacy, the more likely farmers are to become entrepreneurs (Wang et al., 2019). Sequeira et al. (2007) showed that personal social networks together with entrepreneurial self-efficacy contribute to entrepreneurial intentions and entrepreneurial behavior. Wilson et al. (2009) showed that entrepreneurial self-efficacy has a significant impact on entrepreneurial interest and career choice. Kasouf et al. (2015) showed that social capital and human capital contribute to entrepreneurial self-efficacy, and entrepreneurial self-efficacy positively influences entrepreneurial behavior. High risk-bearers have higher entrepreneurial intentions, which can trigger entrepreneurial behavior. Based on the fact that entrepreneurial self-efficacy is a key factor that triggers potential entrepreneurs to implement entrepreneurial behaviors, scholars have proposed different research models by combining the characteristics of farmers. The relationship between entrepreneurial self-efficacy and entrepreneurial behavior through theoretical research, and concluded that the study of entrepreneurial self-efficacy can provide an in-depth understanding of entrepreneurial behavior and has a positive effect on improving the entrepreneurial environment (Chen et al., 1998). Tantawy et al. (2021) showed that entrepreneurial self-efficacy influences farmers’ entrepreneurial decisions by affecting entrepreneurial intentions, leading to entrepreneurial behaviors. This shows that entrepreneurial self-efficacy has a facilitating effect on entrepreneurial behavior. Accordingly, the following research hypothesis is proposed:

**Hypothesis (H3).** Entrepreneurial attitude is positively related to entrepreneurial self-efficacy.
**Hypothesis (H4).** Entrepreneurial environment is positively related to entrepreneurial self-efficacy.
**Hypothesis (H5).** Entrepreneurial self-efficacy is positively correlated with entrepreneurial behavior.

### Conceptual Model

**FIGURE 1:**
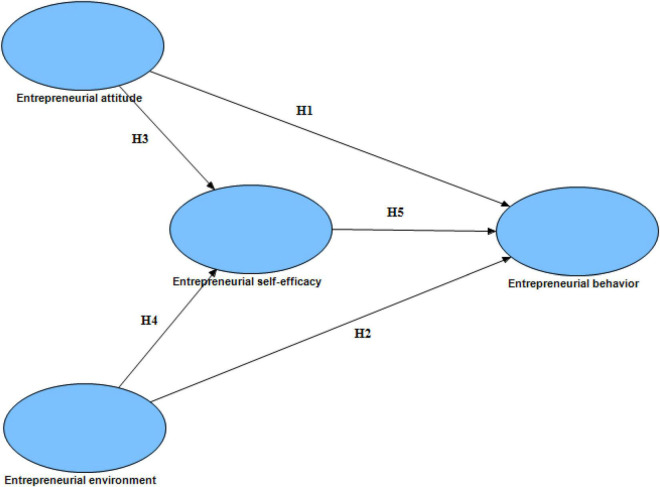
Conceptual model.

Based on the above hypotheses, a conceptual model of the relationship between the four latent variables of entrepreneurial attitude, entrepreneurial environment, entrepreneurial self-efficacy, and entrepreneurial behavior was constructed, as shown in Figure 1.

## Materials and Methods

### Sampling and Data Collection

The scope of this survey was concentrated in the capital regions of the five western provinces. Farmers in Xi’an Xining, Yinchuan, Lanzhou, and Urumqi are located in special geographical locations. On the one hand, as the capitals of cities in western China, the five regions have relatively rapid economic development and a large proportion of the population with high incomes. However, the income of farmers in the suburbs is still not high, so there will be serious income inequality, which is not conducive to the development of a harmonious society. Entrepreneurship becomes a means of adjustment; on the other hand, the provincial capital city has a superior location and convenient transportation and commerce. Entrepreneurship brings certain advantages. Therefore, it is necessary and feasible to study the entrepreneurial behavior of farmers in the five western provinces.

The survey time is from June to July 2019. The questionnaire was answered by the most widely used questionnaire survey website “Questionnaire Star” in mainland China, and sampling was completed through the Ministry of Agriculture and Rural Affairs and the Shaanxi Provincial State-owned Assets Supervision and Administration Commission. This research takes farmers from five western provinces as the research object. The research area covers Chang’an District and Huyi District of Xi’an City; Xixia District and Xingqing District of Yinchuan City; Honggu District and Chengguan District of Lanzhou City (Six inner suburbs); West District and Chengbei District of Xining City; Shuimogou District and Toutunhe District of Urumqi City (Four outer suburbs). The total number of survey samples is 1000 (“Entrepreneurship” and “Unemployment” are designed according to the ratio of 3:7), and the regional distribution of the sample is determined according to the proportion of the population. After the number of regional samples is determined, random sampling is performed and the questionnaire is filled in with the network ad-dress According to the level from district to sub-district (town/township), to the village, and finally to the survey respondents. The survey received a total of 757 questionnaires, with a recovery rate of 75.7%. According to the research design, this research mainly focuses on “start-up” farmers. The number of valid questionnaires is 292. The regional distribution of the 292 questionnaires is shown in Table 1.

**TABLE 1:** Distribution of questionnaires on entrepreneurship of rural farmers in the five western provinces.

Inner suburbs	Number of survey subjects (persons)	Number of entrepreneurial farmers (person)	Outer suburbs	Number of survey subjects (persons)	Number of entrepreneurial farmers (person)
Chang’an District	110	26	West District	24	8
Huyi District	97	36	Chengbei District	28	13
Xixia District	29	16	Shuimogou District	20	12
Xingqing District	141	84	Toutunhe District	25	20
Honggu District	190	36			
Chengguan District	93	41			
Sum	660	239	Sum	97	53

Descriptive analysis was used to characterize and understand the status of the survey sample. Descriptive statistics included age, gender, level of education, and income level. The response group consisted of 147 (50.3%) female respondents and 145 (49.7%) male respondents. The population aged 19–25 accounted for 14%, those aged 26–35 accounted for 60.7%, those aged 36–50 accounted for 25.35%; those with a junior high school education and below accounted for 2.1%, high school and technical secondary school accounted for 15.4%, junior college and Bachelor degree accounted for 78.1%, master degree or above accounted for 4.5%; married people accounted for 75.7%, un-married people accounted for 22.6%, divorced accounted for 1.7%; family monthly income of less than 10,000 yuan accounted for 0.7%, 10,000–20,000 yuan accounted for 1.7%, 20,000–50,000 yuan accounted for 18.2%, 50,000–100,000 yuan ac-counted for 23.3%, 100,000–150,000 yuan accounted for 16.1%, 150,000–200,000 yuan accounted for 13.4%, 200,000–250,000 yuan accounted for 9.6%, 250,000–300,000 yuan accounted for 4.1%, 300,000 yuan or more accounted for 13.0%; the number of people with permanent residence in the city accounted for 59.2%, the permanent city, no urban household registration accounted for 32.9%, and the immigrant population accounted for 7.9%.

### Measures

The article contains four variables: entrepreneurial attitude, entrepreneurial environment, entrepreneurial self-efficacy, and entrepreneurial behavior. The Likert 7-level scale is used to measure the above variables, and the measurement range is from “very dissatisfied” to “very satisfied” corresponding to the numbers “1” to “7.”

The design of the scale mainly draws on the existing mature scale and modified it under the characteristics of entrepreneurial farmers in the suburbs (suburbs) of cities in the five western provinces. Among them, the entrepreneurial attitude is modified by the characteristics of farmers, and the items are mainly set from the perspectives of cognition and evaluation of entrepreneurship; the entrepreneurial environment measurement scale is mainly combined with Eckert (1999) on the impact of entrepreneurial environment (Eckert, 1999). The research on the impact of entrepreneurial behavior is designed into a scale based on the characteristics of suburban farmers, which is mainly measured from five dimensions: policy support environment, socio-economic environment, technological and cultural environment, financial service environment, and infrastructure environment. For the measurement items of different dimensional variables of the entrepreneurial environment, the item with the highest validity in each dimension is selected as the scale; the entrepreneurial self-efficacy measurement scale mainly combines Armitage and Conner (1999) on entrepreneurship The article on the study of the dimension of self-efficacy is modified under the characteristics of suburban farmers and is mainly measured from the five dimensions of innovation efficiency, risk tolerance, opportunity identification, relationship coordination, and organizational commitment (Armitage and Conner, 1999). For the measurement items of different dimensional variables of entrepreneurial self-efficacy, the item with the highest validity among the variables of each dimension is selected as the scale; entrepreneurial behavior is mainly combined with the article by Ilouga et al. (2020) on the measurement of the dimensional structure of entrepreneurial behavior in industrial clusters. According to the characteristics of suburban farmers, the entrepreneurial behavior is divided into five dimensions in consideration of the different stages of the implementation of entrepreneurial behavior: entrepreneurial opportunity identification, entrepreneurial team formation, entrepreneurial resource integration, entrepreneurial network construction, and imitation behavior (Ilouga et al., 2020). For the measurement items of different dimensions of entrepreneurial behavior, the item with the highest validity among the variables of each dimension is selected as the scale.

## Analysis Results

The article uses the structural equation model (SEM) to study the direct and indirect complex relationships between farmers’ entrepreneurial behavior and entrepreneurial attitude, entrepreneurial environment, and entrepreneurial self-efficacy in the suburban areas of the five western provinces (Hair et al., 2011). The state-of-the-art partial least squares structural equations (PLS-SEM) were not used in this study because of: (1) lack of consistency results; (2) lack of model fit metrics (Henseler et al., 2014). Structural equation modeling is a multivariate statistical technique that combines factor analysis and path analysis (Podsakoff et al., 2003; Hair et al., 2012). It can handle the relationship between multiple causes and multiple results in a system and make up for the shortcomings of traditional statistical methods (Hair et al., 2019). It is an important tool for multivariate data analysis.

### Reliability and Validity Test

The scale used in the article draws on a relatively mature scale and is modified based on the characteristics of farmers in the suburbs of the five western provinces. To ensure that the scale is reliable and effective, and the follow-up empirical analysis results are true and meaningful, the reliability and validity of the scale are tested first.

Reliability analysis is an indicator to evaluate the consistency and stability of measurement results. The article uses the α reliability coefficient method for reliability analysis. By analyzing the size of the Cronbach’s α coefficient, it is determined whether the measurement scale has credibility, and the combined reliability (CR) is tested to test whether there is internal consistency between the observed variable and the latent variable. The overall Cronbach’s α coefficient of the scale is greater than 0.7, indicating that the overall reliability of the scale is high. The Cronbach coefficient of each variable is greater than 0.7, indicating that the reliability of each variable is good, and the CR is greater than 0.6, indicating that the observed variable and the latent variable there is internal consistency. After reliability testing, it is showed that the reliability of each variable and the overall reliability are greater than 0.8 (>0.7), the overall Cronbach’s α coefficient is 0.940, and the CR is greater than 0.6, indicating that the measurement results are stable, consistent and credible. The specific values are shown in Table 2.

**TABLE 2:** Test results of reliability and validity of variables.

Variables and measurement questions	Standard load	Average extracted variance	CR
**Entrepreneurship attitude (α = 0.812)**		0.508	0.804
In our place, most of the entrepreneurs have a very good life	0.640		
Entrepreneurship is not difficult	0.794		
Compared with full-time work, entrepreneurship makes family life happier	0.688		
Having entrepreneurial experience will make it easier for me to work in the future	0.719		
**Entrepreneurship environment (α = 0.895)**		0.585	0.874
The government will provide entrepreneurial projects	0.801		
The local economy is developing very fast	0.641		
Talent training will be held locally	0.830		
There are a variety of local financing channels to choose from	0.862		
There are many natural resources for entrepreneurship in the local area	0.665		
**Entrepreneurship self-efficacy (α = 0.905)**		0.480	0.822
I often put forward new ideas and suggestions	0.692		
I like adventure	0.651		
I am good at analyzing the external environment and discovering potential problems	0.731		
I have the confidence to solve the difficulties encountered in the process of interacting with others	0.665		
In order to achieve the goal, I started actual preparations	0.723		
**Entrepreneurship behavior (α = 0.885)**		0.538	0.852
I can well identify valuable market opportunities	0.622		
I can let everyone start a business with one heart	0.795		
When starting a business, I can actively look for new resources to make up for the lack of existing resources	0.792		
I attach great importance to establishing good relationships with technical service teams, technical experts, etc.	0.812		
I tend to learn from the management experience and system of successful entrepreneurs	0.619		

Validity analysis is a test of how close the measured value is to the actual value. The higher the validity, the more consistent the measurement result is with the actual value. The article uses factor analysis to analyze the validity of the scale and determines whether the measurement results of the scale are highly consistent with the actual values by analyzing the factor loading of each item. The factor analysis first needs to judge the applicability, and then analyze the content validity and structure validity separately to complete the validity test of the scale.

First, the applicability of factor analysis is verified by the Bartlett Ball degree test and KMO test. Relevant research shows that when the Bartlett Ball degree test is significant and KMO > 0.8, the method of factor analysis is applicable. After research, it is showed that the Bartlett Ball degree test is significant, and KMO = 0.932 (>0.8), indicating that the method of factor analysis is applicable. Then, the validity test is carried out to test the content validity and structural validity of the scale, respectively. Because each item of the scale refers to the existing mature scale, it has good content validity. When the factor loading is greater than 0.6, the average extracted variance value (AVE) is greater than 0.5, and the KMO value is greater than 0.7, it indicates that the scale has good structural validity. In the article, each variable factor load satisfies greater than 0.6 (see Table 2). The average extracted variance is greater than 0.5. Only the average extracted variance of entrepreneurial self-efficacy is 0.480, but the combined reliability of this variable is 0.822, which is still It can be considered that it has good convergent validity, and the KMO value is 0.932 (>0.8), which all meet the conditions, indicating that the convergent validity is good, and the validity of the scale has passed the test.

### Structural Equation Model Test

**FIGURE 2:**
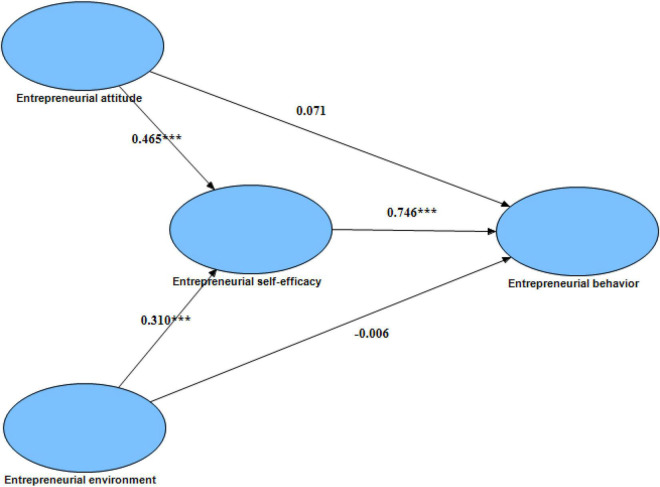
Structural equation model path diagram. *** means significant at the 0.001 level.

After the reliability test and the validity test are passed, SPSS and AMOS are used to analyze the path of the model. Import the data imported from SPSS into AMOS, and after model correction, the common method deviation is eliminated. The final fitness index is shown in Table 3. By observing the index fit table, it is showed that the model and the sample fit well. The path fit results are shown in Table 4, and the standardized path coefficient diagram is shown in Figure 2. It can be showed that the hypotheses H1 and H2 have not passed the test, and the hypotheses H3, H4, and H5 have passed the test. The results show that entrepreneurial attitude and entrepreneurial environment cannot directly promote entrepreneurial behavior, and need to indirectly promote entrepreneurial self-efficacy as an intermediate variable.

**TABLE 3:** Index fit.

	Absolute adaptation index			Incremental fit index					Simple adaptation index			
Testing statistic	RESEA	GFI	AGFI	NFI	RFI	IFI	TLI	CFI	PGFI	PNFI	PCFI	X^2^/df
Fit standard	<0.08	>0.90	>0.90	>0.90	>0.90	>0.90	>0.90	>0.90	>0.50	>0.50	>0.50	<5.00
Verify the results	0.045	0.934	0.902	0.949	0.932	0.980	0.974	0.980	0.629	0.710	0.734	1.582

**TABLE 4:** SEM related path inspection index.

	Estimate	S.E.	C.R.	*P*	Pass or fail
Entrepreneurial self-efficacy ← Entrepreneurial attitude	0.465	0.069	6.727	***	Pass
Entrepreneurial self-efficacy ← Entrepreneurial environment	0.310	0.061	5.056	***	Pass
Entrepreneurial behavior ← Entrepreneurial attitude	0.071	0.067	1.063	0.288	Fail
Entrepreneurial behavior ← Entrepreneurial environment	−0.006	0.051	−0.120	0.950	Fail
Entrepreneurial behavior ← Entrepreneurial self-efficacy	0.746	0.085	8.758	***	Pass

*** Means significant at the 0.001 level; ** means significant at the 0.01 level; * means significant at the 0.05 level.

### Mediating Effect Analysis

The mediating effect of entrepreneurial self-efficacy is analyzed according to the mediating effect test procedure proposed by Maitlo et al. (2020). First, divide the path into two groups, and then regress the independent variable X and the dependent variable Y, the independent variable X and the intermediate variable M, the independent variable X and the intermediate variable M and the dependent variable Y, and test the significance level of the regression coefficient. The results are shown in Table 5.

**TABLE 5:** Mediating effect test.

Standardized regression equation	Regression coefficients	*t*-Value	Significance	ab/c
Entrepreneurial Attitude (*X*_1_)—Entrepreneurial Self-Efficacy (*M*)—Entrepreneurial Behavior (*Y*)				0.773
*Y* = *cX*_1_ + *e*_1_	*c* = 0.562	11.560	0.000	
*M* = *aX*_1_ + *e*_2_	*a* = 0.627	13.694	0.000	
*Y* = *c*′*X*_1_ + *bM* + *e*_3_	*b* = 0.693	14.653	0.000	
	*c*′ = 0.127	2.691	0.008	
Entrepreneurial Environment (*X*_2_)—Entrepreneurial Self-Efficacy (*M*)—Entrepreneurial Behavior (*Y*)				0.767
*Y* = *cX*_2_ + *e*_1_	*c* = 0.547	11.122	0.000	
*M* = *aX*_2_ + *e*_2_	*a* = 0.603	12.866	0.000	
*Y* = *c*′*X*_2_+*bM*+*e*_3_	*b* = 0.696	15.080	0.000	
	*c*′ = 0.127	2.751	0.006	

The regression results show that in the two paths, the total effect c of the independent variable X on the dependent variable Y is significant, the regression coefficients a and b are tested in turn, and the indirect effect is significant; the regression coefficient c’ is tested, and the intermediate variable M is controlled. After the influence of, the direct effect of the independent variable X on the dependent variable Y is significant; a, b, and c’ have the same sign, and the mediation effect is established. And in these two sets of path relationships, the mediating effect accounted for 77.3% and 76.7% of the total effect, respectively. It can be seen that entrepreneurial self-efficacy plays a very important role in promoting entrepreneurial behavior.

## Discussion and Conclusion

### Theoretical Contribution

The results of the empirical study showed that hypotheses H3, H4, and H5 were verified, while hypotheses H1 and H2 were not. The conclusions can thus be summarized into three points.

First, Farmers’ entrepreneurial attitudes cannot directly influence entrepreneurial behavior; entrepreneurial attitudes need to act indirectly on entrepreneurial behavior through entrepreneurial self-efficacy, which plays a mediating role. On the one hand, entrepreneurial attitudes can promote entrepreneurial behavior through entrepreneurial self-efficacy. When farmers think that entrepreneurship is valuable and not difficult, i.e., they have positive entrepreneurial attitudes, they will have interest and confidence in entrepreneurship and confidence in entrepreneurship and have confidence in solving problems in the process of entrepreneurship by themselves in the future, i.e., they will have a higher sense of entrepreneurial self-efficacy, which will contribute to the generation of entrepreneurial behavior. On the other hand, positive entrepreneurial attitudes do not directly generate entrepreneurial behavior. Because even if there is a positive entrepreneurial attitude, if the local external social environment does not provide favorable entrepreneurial facilities in the process of entrepreneurship, and if the entrepreneur does not have enough confidence in solving entrepreneurial problems by himself, i.e., does not have a high entrepreneurial self-efficacy, it will not contribute to the generation of entrepreneurial behavior.

Second, the entrepreneurial environment in which farmers live cannot directly influence farmers’ entrepreneurial behavior; the entrepreneurial environment needs to act indirectly on entrepreneurial behavior through entrepreneurial self-efficacy, which plays a mediating role. On the one hand, a favorable entrepreneurial environment can promote entrepreneurial behavior through a sense of entrepreneurial self-efficacy. The entrepreneurial environment includes many dimensions such as policy support, economic development, and social culture. When farmers in the five western provinces are in an atmosphere conducive to entrepreneurship such as policy support, rapid economic development, and a strong social and cultural atmosphere, the convenient environment will provide help to solve the problems encountered on the way to future entrepreneurship, and prompted by personal character traits will make individuals have confidence in themselves and a higher sense of entrepreneurial self-efficacy, thus promoting entrepreneurial behavior. On the other hand, the entrepreneurial environment will not have a direct impact on entrepreneurial behavior. Because the entrepreneurial environment is an external factor, if an individual does not have the willingness to start a business and has a low tolerance for the risks encountered in the process of starting a business, it will not drive individuals to implement entrepreneurial behavior. Therefore, even if the environment is superior, entrepreneurial behavior will not occur.

Third, in the model, entrepreneurial attitude is a personal factor and entrepreneurial environment is an external factor, both of which cannot directly influence entrepreneurial behavior, but indirectly through entrepreneurial self-efficacy. As a special group, farmers themselves know less about entrepreneurship, have less relevant knowledge base and less experience. As a result, they cannot quickly identify potential opportunities in the entrepreneurial process, have lower risk tolerance, and are not capable of agreeing on complex relationships in all aspects of the entrepreneurial process, which results in the immature development of key intrinsic drivers in the entrepreneurial process, and even with a positive entrepreneurial attitude and a favorable entrepreneurial environment created by the government, it does not mean that they will eventually implement entrepreneurial behavior. Thus, the key to driving farmers to implement entrepreneurial behavior becomes the intrinsic drivers that motivate farmers to start their own businesses. These intrinsic drivers make up the different dimensions of entrepreneurial self-efficacy. Based on this, entrepreneurial self-efficacy becomes a key variable to motivate farmers’ entrepreneurship. It has a positive significance in promoting the generation of farmers’ entrepreneurial behavior. Entrepreneurial self-efficacy measures confidence in self-solving in the entrepreneurial process in different dimensions, is a comprehensive variable that integrates personal characteristics and environmental factors, is the result of considering both intrinsic and extrinsic factors together, and is a key variable with a large mediating effect as a proportion of the total effect.

### Management Implications

Based on the above findings, it can be seen that entrepreneurial attitudes and entrepreneurial environment act indirectly on entrepreneurial behavior through entrepreneurial self-efficacy. Entrepreneurship is a dynamic, multifaceted, and complex process that is influenced by a variety of factors, including the internal self and the external environment (Hrytsaienko et al., 2019). Entrepreneurial self-efficacy is an entrepreneur’s self-evaluation and recognition of his or her personal accomplishment of entrepreneurial goals (Dias et al., 2019a,b). This study supports the findings of Sargani et al. (2021a,b), and the results support the design hypotheses and clarify that personal traits play an important role in supporting sustainable entrepreneurship. Therefore, in a complex entrepreneurial context, after taking personal and environmental factors into account, entrepreneurial self-efficacy can be divided into five dimensions from different perspectives, which are innovation efficacy, risk tolerance, opportunity recognition, relationship coordination, and organizational commitment. Among them, innovation efficacy refers to the entrepreneur’s subjective evaluation of self-innovation ability in the process of entrepreneurship. Risk tolerance efficacy refers to the entrepreneur’s self-approval of his or her ability to resolve uncertainties such as conflicts encountered in entrepreneurship. Opportunity recognition efficacy refers to the entrepreneur’s self-approval of grasping market opportunities in the process of entrepreneurship. Relationship coordination efficacy refers to the entrepreneur’s self-approval of coordinating complex interpersonal relationships in entrepreneurship (Kong et al., 2019). Organizational commitment efficacy refers to the entrepreneur’s self-approval of implementing actions and continuing to run the business (Xie et al., 2021). Entrepreneurial attitude and entrepreneurial environment can indirectly act on entrepreneurial behavior through the five different dimensions of entrepreneurial self-efficacy.

**FIGURE 3:**
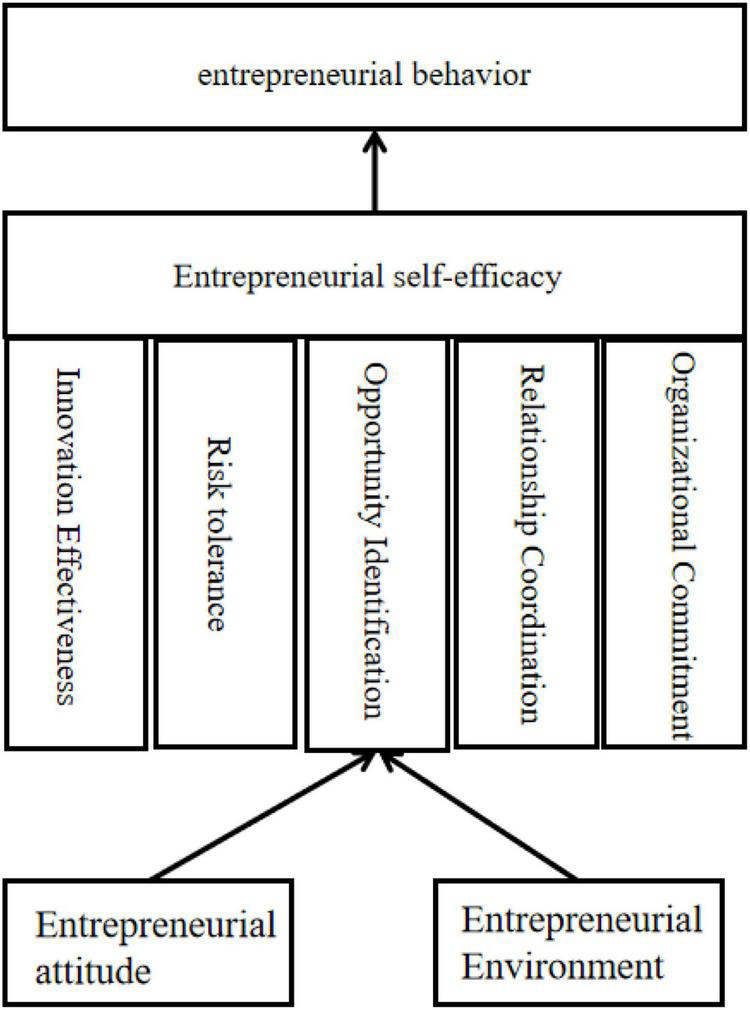
Structural model of entrepreneurial behavior.

In this manuscript, the process of entrepreneurial behavior generation is divided into three levels–primary, secondary, and ultimate. Combining the above statements, a structural model of entrepreneurial behavior is proposed (see Figure 3). The first level, which belongs to the primary level. The factors at this level cover both personal factors - entrepreneurial attitude and environmental factors–entrepreneurial environment. The second level, belongs to the intermediate level. This level covers entrepreneurial self-efficacy–the result of both personal and environmental factors. The third level covers, belongs to the ultimate level, and this level covers entrepreneurial behavior–the behavior that the entrepreneur ultimately implements. “The Structural Model of Entrepreneurial Behavior” indicates that fostering entrepreneurial attitudes and creating an entrepreneurial environment do not directly contribute to farmers’ entrepreneurial behavior, but rather stimulate farmers’ sense of self-efficacy, thus providing a direction for efforts to promote farmers’ entrepreneurial policy formulation and practical guidance. It can be seen that fostering entrepreneurial attitudes and entrepreneurial environment is only the first level of work to promote farmers’ entrepreneurship, and this level of work should promote the formation of farmers’ sense of self-efficacy rather than the direct goal of promoting the generation of agricultural entrepreneurial behaviors. Accordingly, the sense of entrepreneurial self-efficacy formed through the first level of work can effectively promote the formation of farmers’ entrepreneurial behaviors and realize the progression of farmers’ entrepreneurship promotion layer by layer (Arafat et al., 2020).

Therefore, the five dimensions of entrepreneurial self-efficacy can be targeted to specifically develop specific measures to foster entrepreneurial attitudes and create an entrepreneurial environment. Extends the results of Sargani et al. (2020, 2021a) applied Theory of Planned Behavior study. In other words, improving the sense of innovation efficacy, risk tolerance efficacy, opportunity recognition efficacy, relationship coordination efficacy, and organizational commitment efficacy is the focus of formulating innovation and entrepreneurship policies, improving the entrepreneurial environment, and fostering entrepreneurial attitudes.

### Limitations and Future Research

This study solved some gaps in the literature, but there are still some limitations that need further discussion. First, the data used in this study was collected only in China. Since different research Settings and other samples may lead to different findings, researchers may wish to use data from other emerging economies to examine various entrepreneurial actions. Secondly, this study can use the fuzzy set qualitative comparative analysis method to study the antecedents of entrepreneurial behavior, and obtain the path of high entrepreneurial behavior.

## Data Availability Statement

The original contributions presented in the study are included in the article/supplementary material, further inquiries can be directed to the corresponding author/s.

## Author Contributions

HD and BW: methodology, software, and formal analysis. XC: resources and data curation. HD and PZ: investigation. HD: writing—original draft preparation, supervision, and project administration. HD and XC: writing—review and editing. JH: significant revisions to the research hypotheses in the full text were proposed, and comments were provided to support the validation of the methodological analysis. JH also coordinated the subject fund support. All authors have read and agreed to the published version of the manuscript.

## Conflict of Interest

HD, BW, PZ, and JH were employed by Shaanxi Provincial Land Engineering Construction Group Co., Ltd. The remaining author declares that the research was conducted in the absence of any commercial or financial relationships that could be construed as a potential conflict of interest.

## Publisher’s Note

All claims expressed in this article are solely those of the authors and do not necessarily represent those of their affiliated organizations, or those of the publisher, the editors and the reviewers. Any product that may be evaluated in this article, or claim that may be made by its manufacturer, is not guaranteed or endorsed by the publisher.
